# The Development of Love Wave-Based Humidity Sensors Incorporating Multiple Layers

**DOI:** 10.3390/s150408615

**Published:** 2015-04-14

**Authors:** Lijun Wang, Jiansheng Liu, Shitang He

**Affiliations:** Institute of Acoustics, Chinese Academy of Sciences, Beijing 100190, China; E-Mails: wanglijun12@mails.ucas.ac.cn (L.W.); heshitang@mail.ioa.ac.cn (S.H.)

**Keywords:** Love wave, humidity sensor, viscoelastic, multiple layers

## Abstract

A Love wave humidity sensor is developed by using a multilayer structure consisting of PVA/SiO_2_ layers on an ST-90°X quartz substrate. The theoretical result shows that the sensor with such a two-layer structure can achieve a higher sensitivity and a smaller loss than the structures with a single polymer layer. Comparative experiments are performed for the sensor incorporating PVA/SiO_2_ layers and the sensor incorporating a PVA layer. The experimental results agree well with the theoretical predication.

## 1. Introduction

Humidity detection plays an important role in a variety of commercial and industrial applications like process monitoring in the chemical industry, agricultural production, meteorology, *etc.* [1]. Due to their high sensitivity to the surface mass loading phenomenon, recently a growing number of surface acoustic waves (SAWs) techniques [2,3,4,5] have been employed for various sensor applications, wherein SAW humidity sensors have attracted much attention. A Love wave is a kind of layered SAW with particle displacement only in the shear horizontal (SH) direction. Due to the guiding layer, the energy of a Love wave is concentrated in the guiding layer and the substrate near the surface; thus it is very sensitive to any disturbance loading on the surface of the guiding layer and this feature can be used to produce a sensor with good performance features such as high sensitivity, adjustable temperature coefficient, and small coupling loss in liquids, *etc.* Therefore Love wave sensors have been attracting the interest of many researchers since they were first reported in 1992 [6,7]. Different from the commonly used Rayleigh type SAW, the properties of Love waves depend on the guiding layer rather than the interdigital transducer (IDT) structure and substrate piezoelectricity. Elastic layers such as SiO_2_ have the merits of low acoustic loss and excellent abrasion resistance; however a sensor coated a SiO_2_ layer cannot achieve a very high sensitivity because of its fast transverse wave. Polymers have slower transverse waves and lower density, so they are often used as guiding layers for Love wave sensors to achieve a higher sensitivity. Unfortunately, the viscosity of polymers can produce a great propagation loss. To get a balanced good performance, some researchers prefer sensors with two layers to combine the advantages of both elastic and polymeric layers.

Du *et al.* [8,9,10,11,12] reported a Love wave sensor with a hybrid structure of a PMMA and a SiO_2_ layers on an ST-quartz substrate. The results show that such a structure can achieve a more stable temperature performance, a larger mass loading sensitivity, and an acceptable insertion loss. McHale *et al.* [13,14] developed a method for describing Love wave and SH-APMs sensors with multiple viscoelastic guiding layers. In their studies, a Maxwell model was adopted to describe the viscoelasticity of the polymeric layers or the liquids above the layer. Recently the authors [15,16,17,18] also presented a theoretical model for analyzing the performance of Love wave sensors based on a structure with multiple viscoelastic or elastic guiding layers on a piezoelectric substrate. The results prove that a Love wave sensor with such a two-layer structure can achieve better performances than with only one viscoelastic or elastic guiding layer. However, until now, no gas experiment research on this double waveguide layers structure has been reported, especially no experimental research contrasting between this hybrid structure and a monolayer polymer waveguide structure has been reported.

In this work, we present a Love wave humidity sensor based on a device consisting of PVA/SiO_2_ layers on an ST-90°X quartz substrate. The theoretical model for Love waves in the structure with multiple layers on a piezoelectric substrate is briefly reviewed. The viscoelasticity of the PVA layer is described by the Maxwell-Weichert model which was reported in our previous studies. The experimental humidity sensor is implemented based on a Love wave device incorporating a polyvinyl alcohol (PVA) layer and a SiO_2_ layer on an ST-90°X quartz substrate. A comparison experiment using a sensor based on a device incorporating only a PVA layer is also presented. Humidity experiments show that the sensor with the composite layer has a greater shift in operation frequency and a less insertion loss increase. The result indicates that a coated double layer Love wave sensor has more valuable application prospects than a sensor coated with a single layer.

## 2. Theoretical Analysis

### 2.1. Love Waves in a Two Layer Coated Structure 

**Figure 1:**
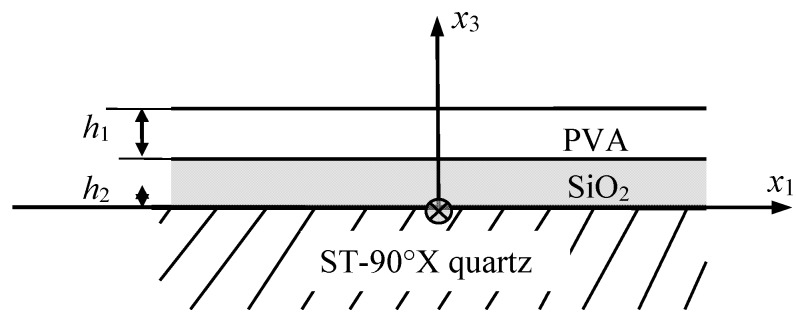
Schematic of the considered Love wave device and coordinate system.

As shown in Figure 1, the Love wave device used in this work consists of two layers on a piezoelectric substrate. The coordinate system is chosen in such a way that the *x*_1_-axis is in the direction of wave propagation and lying along the surface of the substrate, while the *x*_2_-axis is in the direction of particle displacement, and the *x*_3_-axis is vertically upwards. The piezoelectric substrate occupies the half space of *x*_3_ < 0; the SiO_2_ layer occupies the space of 0 < *x*_3_ < *h*_2_; the PVA layer occupies the space of *h*_2_ < *x*_3_ < *h*_1_ + *h*_2_; the space above the PVA layer is filled by the test gas.

The substrate of a Love device must be a piezoelectric material in which the electric field is only coupled with the particle motion in the shear horizontal (SH) direction. The dispersion equation of Love waves in a structure with two layers on a piezoelectric substrate can be written as [15]:

(1) $$\mu_{L2}\beta_{L2}\frac{\mu_{L2}\beta_{L2}\tan\left(k\beta_{L2}h_{2}\right)+\mu_{L1}\beta_{L1}\tan\left(k\beta_{L1}h_{1}\right)}{\mu_{L2}\beta_{L2}-\mu_{L1}\beta_{L1}\tan\left(k\beta_{L1}h_{1}\right)\tan\left(k\beta_{L2}h_{2}\right)}=\frac{\left(D_{2}-\bar{\varepsilon_{L2}}\right)T_{1}-\left(D_{1}-\bar{\varepsilon_{L2}}\right)T_{2}}{\left(D_{2}-\bar{\varepsilon_{L2}}\right)A_{1}-\left(D_{1}-\bar{\varepsilon_{L2}}\right)A_{2}}$$

where *k* is the wave number; β*L*1 and β*L*2 are the transverse propagation constants in each layers respectively; μ*L*1 and μ*L*2 are the transverse modulus respectively; *D*1 and *D*2 are the components of normal electric displacement at the substrate surface; *T*1 and *T*2 are the components of normal stress acting on the surface particle of the substrate; $\bar{\varepsilon_{L2}}$ is the equivalent permittivity of the guiding layers. 

Supposing on the surface of the top layer there is a tiny mass load with an areal density of Δ*m*, we can get the dispersion Equation [15]:

(2) $$\mu_{L2}\beta_{L2}\frac{\mu_{L2}\beta_{L2}\tan\left(k\beta_{L2}h_{2}\right)+\mu_{L1}\beta_{L1}\tan\left[k\beta_{L1}\left(h_{1}+\frac{\Delta mv^{2}}{\mu_{L1}\beta_{L1}^{2}}\right)\right]}{\mu_{L2}\beta_{L2}-\mu_{L1}\beta_{L1}\tan\left[k\beta_{L1}\left(h_{1}+\frac{\Delta mv^{2}}{\mu_{L1}\beta_{L1}^{2}}\right)\right]\tan\left(k\beta_{L2}h_{2}\right)}=\frac{\left(D_{2}-\bar{\varepsilon_{L2}}\right)T_{1}-\left(D_{1}-\bar{\varepsilon_{L2}}\right)T_{2}}{\left(D_{2}-\bar{\varepsilon_{L2}}\right)A_{1}-\left(D_{1}-\bar{\varepsilon_{L2}}\right)A_{2}}$$

The mass load will cause a velocity perturbation of Δ*v*, which is the difference in velocities decided by the dispersion Equations (1) and (2). If both layers are elastic materials with real shear modulus, using the equations we will get velocities of real numbers. A real velocity means that Love wave propagates without attenuation. If some layer is a viscoelastic material, the dispersion equations are also applicable but the shear modulus of the viscoelastic layer becomes a complex number. The complex shear modulus can be described by using the Maxwell-Weichert model consisting of many parallel dashpots and springs branches. In our previous research [16], the model was simplified as two parallel branches, one is an elastic branch consisting of a spring; another is a Maxwell branch consisting of a spring and a dashpot in serial. The complex shear modulus can be expressed as $\mu =\mu_{0}+\frac{i\omega \eta_{1}}{1+i\omega \eta_{1}/\mu_{1}}$, which will result in a complex velocity of *v* = *v_r_* + *iv_i_*. The real part represents the speed of Love waves, and the imaginary part is related to the propagation attenuation. The mass sensitivity also becomes a complex number:

(3) $$S_{mv}=\frac{1}{v_{0}}{\left(\frac{\Delta v}{\Delta m}\right)}_{\Delta m\to 0}=S_{m}^{v_{r}}-i\frac{S_{m}^{IL}}{54.6}$$

where $S_{m}^{v_{r}}$ mass velocity sensitivity of a LW sensor incorporating a viscoelastic guiding layer, $S_{m}^{IL}$ is the mass insertion loss sensitivity. 

**Figure 2:**
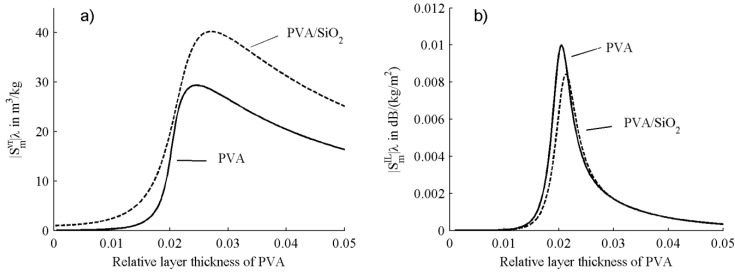
Mass velocity sensitivities (**a**) and mass loss sensitivities (**b**) of Love sensor incorporating a single PVA layer (solid lines) and PVA/SiO_2_ layers (dashed lines).

In Figure 2, the normalized mass sensitivities of a Love wave sensor coated a PVA layer are displayed in comparison with a sensor coated PVA/SiO_2_ layers. The substrate is set as ST-cut 90°X-propagate quartz, whose material constants are obtained from the constants of quartz [19] via a Bond conversion (Eulerian angles of (0°, 132.75°, 90°)). The material constants of PVA are set as: ρ = 1250 kg/m^3^, μ_0_ = 0.2 GPa, μ_1_ = 65 MPa, τ_1_ = 0.9 ns, and ε = 3ε_0_, where ε_0_ is the vacuum permittivity. The SiO_2_ is considered as an elastic layer which has a thickness of 5.7%λ and material constants of ρ = 2200 kg/m^3^, μ = 1.74 GPa, and ε = 3.75ε_0_. The solid curves represent the sensitivities of the sensor incorporating a PVA layer; the dashed curves represent the sensitivities of the sensor coated PVA and SiO_2_ layers. As shown in the figure, the mass velocity sensitivity of the sensor incorporating PVA/SiO_2_ layers is obviously larger that the sensor coated only PVA layer; the mass loss sensitivity of the former is slightly less than the latter.

### 2.2. Adsorption Mechanism

When contacting a solid surface, some gas molecules will remain on the solid surface for some time before returning to the gas phase, which is called adsorption. According to the different bonding forces, adsorptions can be divided into physical adsorptions and chemical adsorptions. For most SAW gas sensors, only the physical adsorption is applicable because of its repeatability and reversibility. For a physical adsorption, the gas adsorption amount is decided by the adsorption temperature, relative gas pressure, and the adsorption properties of the solid surface [20,21]:

(4) $$\frac{V}{V_{0}}=\frac{ckx}{\left(1-kx\right)\left[1+\left(c-1\right)kx\right]}$$

where *V* is the volume occupied by the adsorbed gas molecules; *V*_0_ is the volume occupied by the gas molecules corresponding to a saturated monomolecular layer adsorption; *k* is an adjustable constant decided by the nature of the adsorption system, *x* represents the relative pressure *P*/*P*_0_ where *P*_0_ is the saturated vapor pressure at the adsorption temperature; *c* is an adsorption characteristic parameter of the gas on the solid surface.

By combing Equation (4) and Wohljent’s method, the authors introduced in [22,23] the quantitative relation between gas pressure and the frequency shift of SAW sensor:

(5) $$\frac{\Delta f}{\Delta f_{0}}=\frac{ckx}{\left(1-kx)[1+(c-1)kx\right]}$$

where Δ*f*_0_ is the frequency offset of the SAW oscillator caused by a monomolecular layer of absorbed gas molecules covering the detector surface. Equation (5) describes the relationship between the sensor output and gas concentration, which is the theoretical model of the response mechanism for a SAW gas sensor.

## 3. Experimental Verification

### 3.1. Fabrication of Love Wave Devices

The Love wave devices consist of an ST-90°X quartz substrate and two 200 nm thick Al interdigital transducers (IDTs). Each IDT consists of 72 periods of split-electrodes with the periodicity λ = 28 μm (3.5 μm electrodes and spaces). The IDT aperture is 2 mm and the IDT center to center separation is 4 mm. The uncoated device supports an SH wave with the frequency of ~178.6 MHz and the insertion loss of 26 dB.

SiO_2_ thin films are deposited by using Plasma Enhanced Chemical Vapor Deposition (PECVD) technique at a low temperature (<300 °C). The radio frequency glow discharge excites the reaction gas SiH_4_ and N_2_O into the plasma which consists of electrons with high density, high energy and high temperature. The reaction equation is as follows:

SiH_4_ (gas) + 2N_2_O (gas) → SiO_2_ (solid) + 2N_2_ (gas) + 2H_2_ (gas)

The reaction product SiO_2_ is deposited on the substrate surface and the film thickness is controlled by an ellipsometer. Based on the previous researches, the optimum thickness is about 1.6 μm (~5.7% of the acoustic wavelength).

The commercial PVA powder (average degree of polymerization 1750 ± 50, purity 97%) was provided by Beijing Yili Fine Chemicals Co., Ltd. (Beijing, China). PVA powder (30 g) was put into a clean glass baker, then deionized water (600 mL) was injected into the beaker and evenly mixed with the PVA powder. The mixture was first heated to 60 °C and maintained for 40 min; the PVA powder was softened and began to dissolve. Then the mixture was heated to 90 °C and maintained for 50 min, until the PVA powder was completely dissolved. Throughout the dissolution process, the solution has been shaken by a thermo magnetic stirrer. The PVA solution was cooled in air for 3 h and the remaining solution was about 480 mL.

The prepared PVA solution is dense and sticky, so it was diluted with deionized water to increase mobility during spinning. About 5 mL of diluted PVA solution was dropped on the surface of the IDT-fabricated and SiO_2_-coated quartz wafer; then the wafer was rotated for 50 s at a speed of 3000 rpm. To cure the PVA film, the deposited wafer was placed in air at 60 °C for 30 min. The film on the wire pad was removed by using a sharp scalped blade. The PVA layer is about 0.53 μm, which was measured by using an Alpha-step IQ surface profiler (KLA-Tencor, San Jose, CA, USA).

### 3.2. Experimental Setup

**Figure 3:**
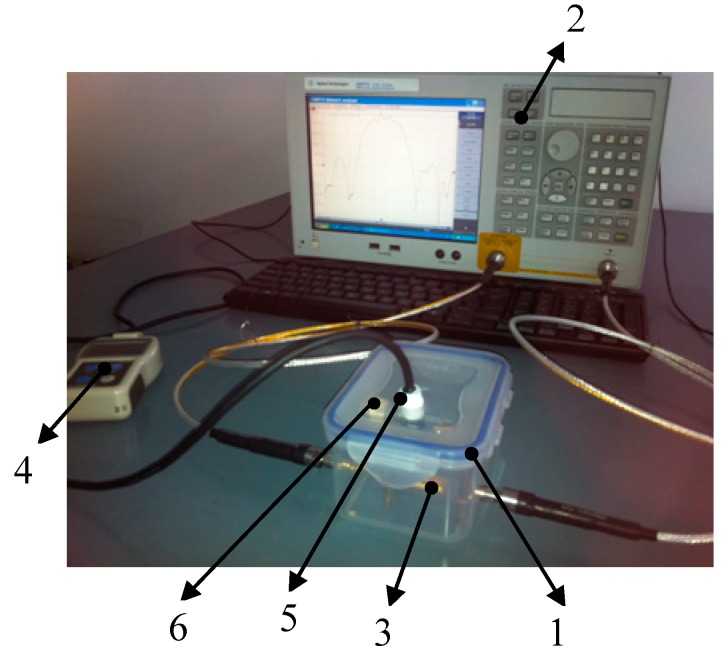
Experimental setup: (1) test box; (2) network analyzer; (3) Love wave device; (4) standard hygrometer; (5) probe of the hygrometer; (6) inlet/outlet for silica particles and wet sponges.

The experimental platform for humidity measurement is shown in Figure 3. The platform consists of a network analyzer (Agilent E5071C, Santa Clara, CA, USA), an RH32B-2C digital thermo-hygrometer (Omega Engineering, Inc. Stamford, CT, USA), and a closed test container. The Love wave device was placed in the plastic container, which has four holes for the connection with the network analyzer, the probe of the standard hygrometer, and wet sponge or silicone particles to change the humidity in the container.

## 4. Results and Discussion

We use silica particles and wet sponges to adjust the humidity in the test box. Silica (*m*SiO_2_·*n*H_2_O) is a kind of porous solid which can strongly adsorb a variety of materials. If we put absorbent silica particles into the test box, the water vapor will be absorbed and the moisture content will be reduced. On the contrary, putting wet sponges will increase the moisture content in the test box. The test humidity can be adjusted by changing the amount of silica particles or wet sponges. The humidity value can be read from the RH32B-2C digital thermo-hygrometer. The temperature is also monitored by using the hygrometer with an accuracy of 0.1 °C, and during the measurement the temperature in the chamber is about 23 °C. When RH = 20%, the device coated only a PVA film works at the frequency of 177.57 MHz and the insertion loss of 8.79 dB; the device coated PVA/SiO_2_ layers works at the frequency of 169.95 MHz and the insertion loss of 8.59 dB.

**Figure 4:**
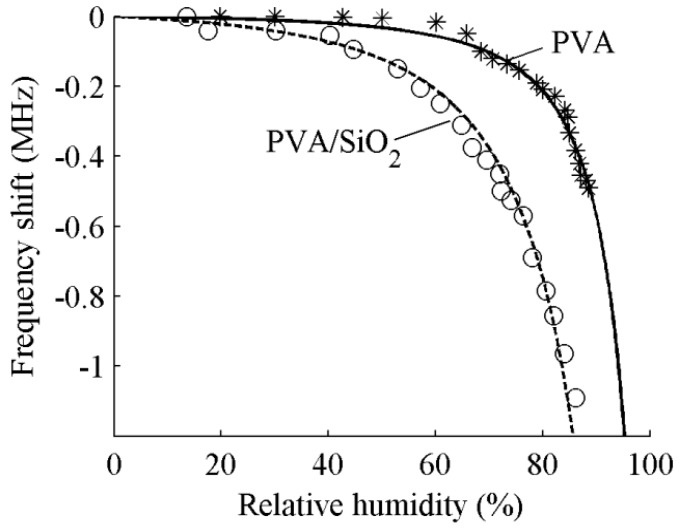
Frequency shifts *versus* relative humidity for ST-90°X quartz substrate/PVA and ST-90°X quartz substrate/SiO_2_/PVA devices.

**Figure 5:**
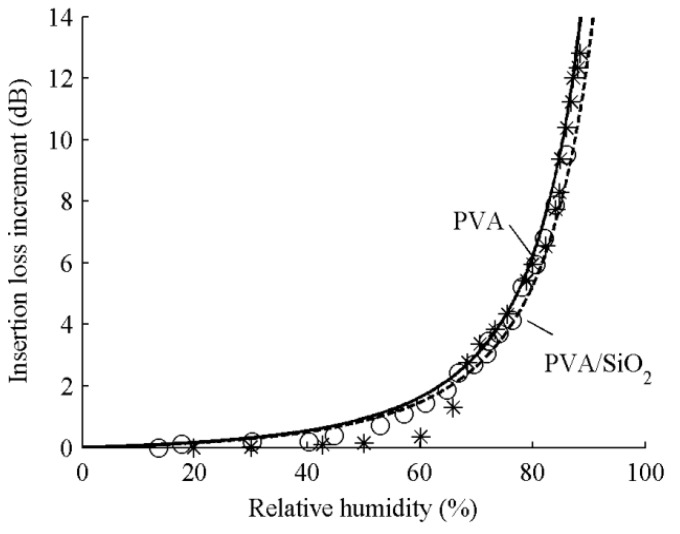
Insertion loss shifts *versus* relative humidity for ST-90°X quartz substrate/PVA and ST-90°X quartz substrate/SiO_2_/PVA devices.

Figure 4 shows the frequency shifts of the sensors incorporating a PVA layer *versus* PVA/SiO_2_ layers. The thickness of PVA film is 0.53 μm for both sensors and the thickness of SiO_2_ film is 1.6 μm for the latter. The asterisks are the measured frequency shifts for the sensor coated a PVA layer and the solid line is calculated by using Equation (4) with the parameters of *k* = 0.96, Δ*f*_0_ = −0.2 MHz and *c* = 0.1. The circles are the measured frequency shifts for the sensor coated PVA/SiO_2_ layers and the solid line is calculated by using Equation (4) with the parameters of *k* = 0.94, Δ*f*_0_ = −0.8 MHz and *c* = 0.1. Figure 5 shows the insertion loss increments of the sensors incorporating a PVA layer *versus* PVA/SiO_2_ layers. The asterisks are the measured insertion loss increments for the sensor coated a PVA layer and the solid line is calculated by using Equation (4) with the parameters of *k* = 0.96, Δ*IL*_0_ = 5.8 dB and *c* = 0.1. The circles are the measured insertion loss increments for the sensor coated PVA/SiO_2_ layers and the solid line is calculated by using Equation (4) with the parameters of *k* = 0.94, Δ*IL*_0_ = 5.6 dB and *c* = 0.1.

As shown in the figures, the same change in humidity causes an obviously frequency shift for the Love wave sensor incorporating PVA/SiO_2_ layers than for the sensor incorporating only a PVA layer. The insertion loss caused by increasing humidity shows a little difference. The result is consistent with the theoretical analysis described in the previous section.

## 5. Conclusions

In this work, a Love wave sensor incorporating polymeric/elastic layers is investigated. Compared to the sensor coated only with a polymeric layer, the sensor with composite layers can achieve a much greater frequency shift and a slightly smaller loss increment. A comparative experiment is performed for a Love wave device incorporating a polyvinyl alcohol (PVA) layer and a SiO_2_ layer on an ST-90°X quartz substrate and a device incorporating only a PVA layer. Humidity experiments show that the sensor coated with two layers can result in an obviously greater shift in operation frequency and a smaller insertion loss increase. The result indicates that a Love wave sensor coated with double layers has more valuable application prospects than a single layer coated sensor. This research will be helpful to develop Love wave sensors with optimized performance and to study the mechanism of SAW sensor response to the humidity content of the test vapor.
